# “Over-the-Top” Anterior Cruciate Ligament Reconstruction Associated With a Lateral Extra-Articular Tenodesis in Children

**DOI:** 10.1016/j.eats.2022.11.026

**Published:** 2023-03-03

**Authors:** Abel Gómez Cáceres, Iskandar Tamimi Mariño, Francisco Javier Martínez Malo, Ignacio Vieitez Riestra, Raphael Pierre Idiart

**Affiliations:** a Hospital HM Málaga, Málaga, Spain; b Málaga Football Club, Málaga, Spain; c Hospital Regional Universitario de Málaga, Málaga, Spain; d Hospital Vithas Xanit Internacional, Málaga, Spain

## Abstract

Anterior cruciate ligament (ACL) tears are one of the most frequent injuries in growing children, and they are often associated with other injuries such as meniscal and chondral injuries. In the past, treatment of ACL tears in growing patients relied on activity modification and bracing. However, surgical treatment has prevailed over conservative treatment in recent years. A surgical technique is presented for ACL reconstruction using an “over-the-top” technique associated with a lateral extra-articular tenodesis procedure in children. An extra-articular lateral tenodesis is done first. The gracilis and semitendinous tendons are then extracted using a tenotome without releasing their distal desinsertions. The tibial guide is then centered over the ACL tibial footprint under arthroscopic vision and an image intensifier, proximal to the physis. Then, a Kocher-type forceps is used to pass a suture “over the top” from the posterolateral window to the tibial tunnel. The double-bundle graft and iliotibial tract graft are fixed within the tunnel in full extension and neutral rotation with an interference screw.

## Technique Video

Video 1 The patient is placed in a supine position with a tourniquet on the proximal thigh. An L-shaped support is placed on the distal end of the table to keep the knee at about 100º of flexion. Two additional supports are placed laterally. An exploration of the knee joint is performed under general anesthesia showing a positive Lachman test. A 3- to 4-cm lateral incision is performed over the lateral epicondyle, and a 1-cm-thick band is detached from the iliotibial band and extended from Gerdy’s tubercle proximally. The free end of the graft is sutured with a high-strength suture and passed under the lateral collateral ligament (LCL). Then, an oblique incision is made on the medial aspect of the tibia. The gracilis and semitendinous tendons are then harvested using a tenotome, without releasing their distal insertions. Next, the tendons are cleaned from any remaining muscle fibers, and their ends are sutured with a high-strength suture. The diameter of the double-bundle autograft is measured to create the tibial tunnel. Standard anterolateral and anteromedial portals are made to perform a full exploration of the knee. The anterior cruciate ligament (ACL) tear is confirmed and a debridement of any remnant tissues is performed using an arthroscopy shaver. The exit point of the tibial tunnel is marked with an electrocautery device*.* The tibial guide is then centered over the ACL tibial footprint under arthroscopic vision and an image intensifier. A guide-pin is inserted proximally to the tibial physis and drilled in an outside-in direction. The angulation of the tibial guide varies depending on the position of the physis. The diameter of the tunnel is determined by the size of the double-bundle graft. Next, a small window is made through the posterolateral capsule of the knee. A Kocher-type forceps is then used to pass a suture “over the top” from the posterolateral window to the anteromedial portal. The suture is then retrieved through the tibial tunnel and used to pass the graft to the posterolateral window. Next, the diameters of the hamstring graft and the iliotibial band graft are measured*.* Finally, a femoral tunnel is drilled proximally and posteriorly to the distal femoral physis under the image intensifier from lateral to medial. The double-bundle graft and iliotibial tract graft are fixed within the tunnel in full extension and neutral rotation with an interference screw.

The number of knee injuries in growing children has increased considerably in recent year due to the higher levels of sports activities and professionalization of sports.^1^ Anterior cruciate ligament (ACL) tears are one of the most frequent injuries in this age group, and they are often associated with other injuries such as meniscal and chondral injuries.^2^

In the past, treatment of ACL tears in growing patients relied on activity modification and bracing.^3^ However, surgical treatment has prevailed over conservative treatment in recent years^4^^,^^5^ since there is a higher risk of chondral and meniscal injuries in patients with ACL deficiency who are treated conservatively.^6^

Regarding the surgical treatment of these injuries, we must bear in mind that these patients are growing. Therefore, the physis should be spared to avoid potential growth deformities. Accordingly, many surgical techniques and treatment algorithms have been described to manage ACL injuries, based on age, sex, and the state of the growth plate. “Over-the-top” techniques with autologous hamstring tendon grafting have been used for the treatment of ACL injuries.^7^^,^^8^

These techniques have few complications involving the growth and angulation of the operated knee, restore joint stability and the biomechanics of the knee, and have a similar reoperation rate and clinical outcomes compared to epiphyseal techniques.^7^^,^^9^^,^^10^

On the other hand, due to their excellent results, lateral reinforcements by associating an extra-articular lateral tenodesis (LET) to ACL reconstruction the “over-the-top” technique is becoming progressively more popular in growing patients with an immature skeleton.^7^^,^^11^

The anterolateral reinforcement associated with reconstruction of the ACL provides greater resistance to internal rotation and anterior tibial translation. It reduces the rate of graft failure, even in patients with a higher risk of failure, such as young patients.^12^, ^13^, ^14^, ^15^

The objective of this study is to report our physis-sparing ACL reconstruction technique using an “over-the-top” technique associated with a lateral extra-articular tenodesis procedure.

## Methods

### Surgical Technique

#### Patient Positioning

**Fig 1:**
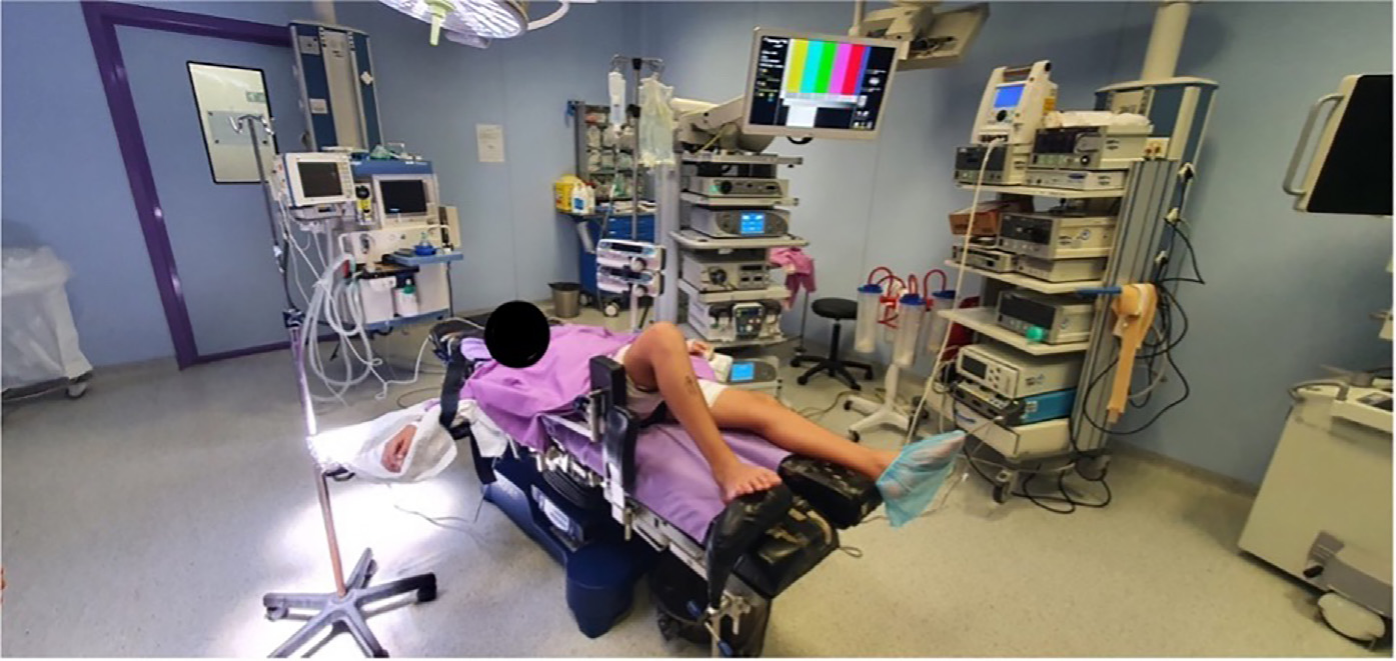
Position of the patient in the operating room. The patient is placed in the supine position. A tourniquet is placed on the proximal thigh, and an L-shaped support is placed on the distal end of the table to keep the knee in about 90° of flexion. Two additional supports are placed laterally.

The patient is subjected to general anesthesia and placed in the supine position. A tourniquet is placed on the proximal thigh, and an L-shaped support is placed on the distal end of the table to keep the knee in about 90º of flexion. Two additional supports are placed laterally (Fig 1, Video 1).

#### Extra-Articular Lateral Tenodesis

**Fig 2:**
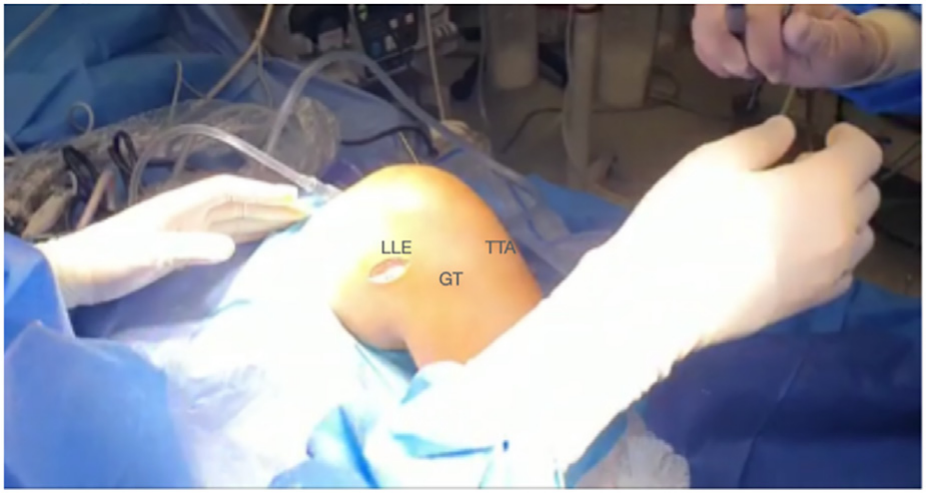
Right knee. A 3- to 4-cm lateral incision is performed over the lateral epicondyle. (GT, Gerdy’s tubercle; LLE, lateral epicondyle; TTA, tuberosity tibial anterior.)

**Fig 3:**
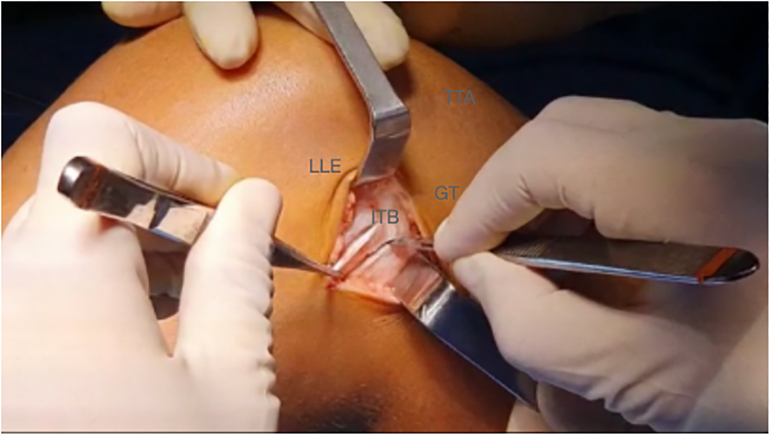
Right knee. In total, 1 cm of the iliotibial tract is detached from Gerdy’s tubercle. It is important to gain the maximum possible length. (GT, Gerdy’s tubercle; ITB, iliotibial band; LLE, lateral epicondyle; TTA, tuberosity tibial anterior.)

**Fig 4:**
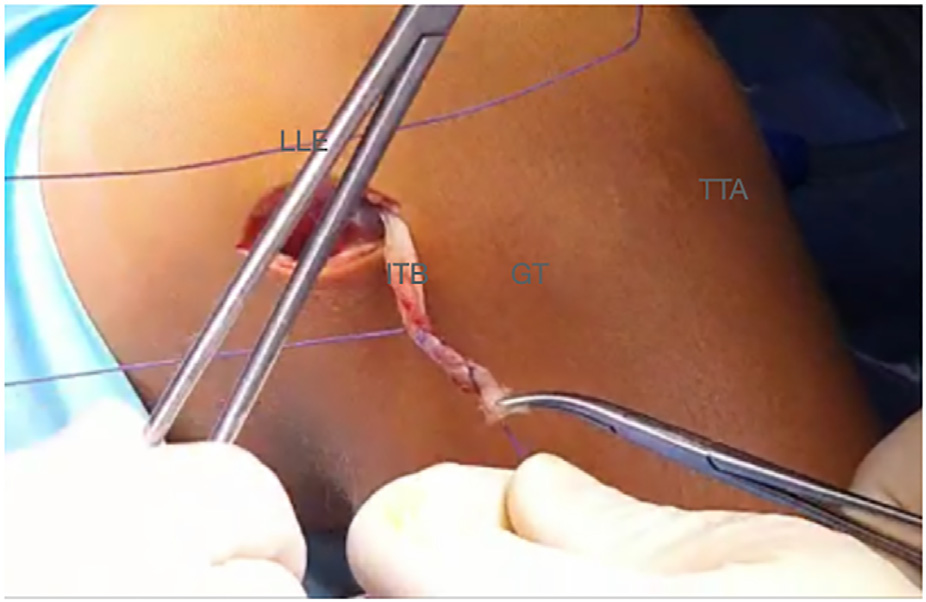
Right knee. The plasty of the iliotibial tract is sutured with a FiberWire (Arthrex) No. 2 suture. (GT, Gerdy’s tubercle; ITB, iliotibial band; LLE, lateral epicondyle; TTA, tuberosity tibial anterior.)

**Fig 5:**
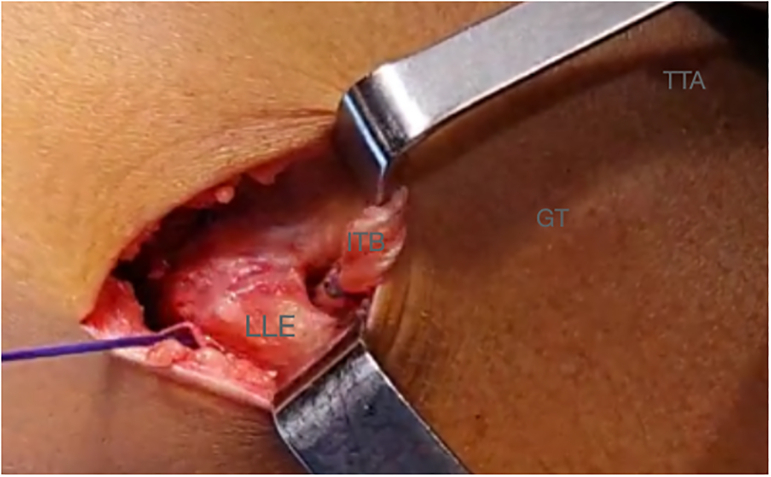
Right knee. The plasty of the iliotibial tract is passed under the lateral collateral ligament (LCL). (GT, Gerdy’s tubercle; ITB, iliotibial band; LLE, lateral epicondyle; TTA, tuberosity tibial anterior.)

A 3- to 4-cm lateral incision is performed over the lateral epicondyle (Fig 2). A 1-cm thick band is detached from the iliotibial band and extended from Gerdy’s tubercle proximally to gain the maximum possible length (Fig 3). The free end of the graft is sutured with a No. 2 high-resistance FiberWire (Arthrex) suture and passed under the lateral collateral ligament (Figs 4 and 5, Video 1).

#### Arthroscopic Exploration

Standard anterolateral and anteromedial portals are made to perform a full exploration of the knee. Then any meniscal injuries are identified and repaired using Truespan (Depuy-Synthes) implants. The ACL tear is confirmed and a debridement of any remnant tissues is performed using a No. 4 arthroscopy shaver (Depuy-Synthes). Then the exit point of the tibial tunnel is marked with an electrocautery device (Depuy-Synthes) (Video 1).

#### Graft Harvesting and Preparation

**Fig 6:**
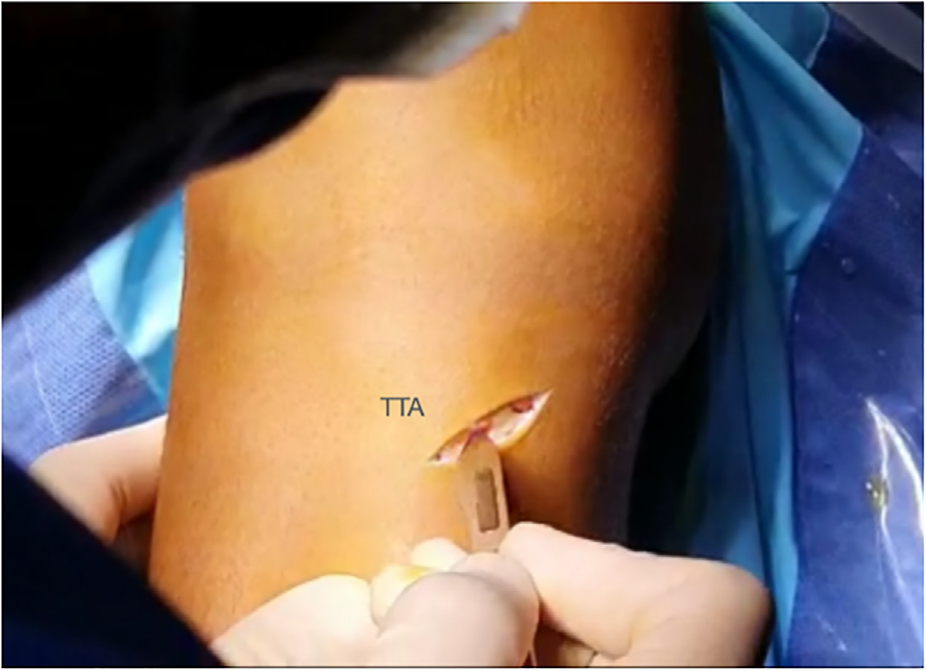
Right knee. Oblique incision on the medial aspect of the tibia for hamstring detachment. (TTA, tuberosity tibial anterior.)

**Fig 7:**
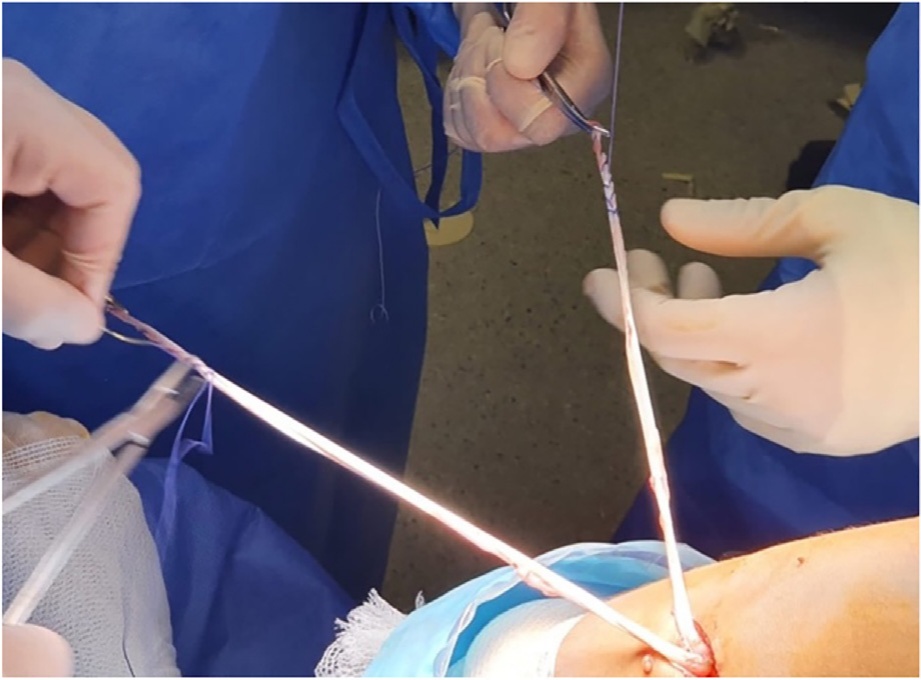
Right knee. The hamstring tendons are sutured independently without releasing their distal deinsertions.

An oblique incision is made on the medial aspect of the tibia, above and in the same direction as the hamstring tendons (Fig 6). The gracilis and semitendinous tendons are then extracted using a tenotome without releasing their distal insertions. Next, the tendons are cleaned from any remaining muscle fibers, and their ends are sutured with a No. 2 high-resistance thread (Fig 7). Finally, the diameter of the double-bundle autograft is measured before drilling the tibial tunnel (Video 1).

#### Tibial Tunnel Preparation

**Fig 8:**
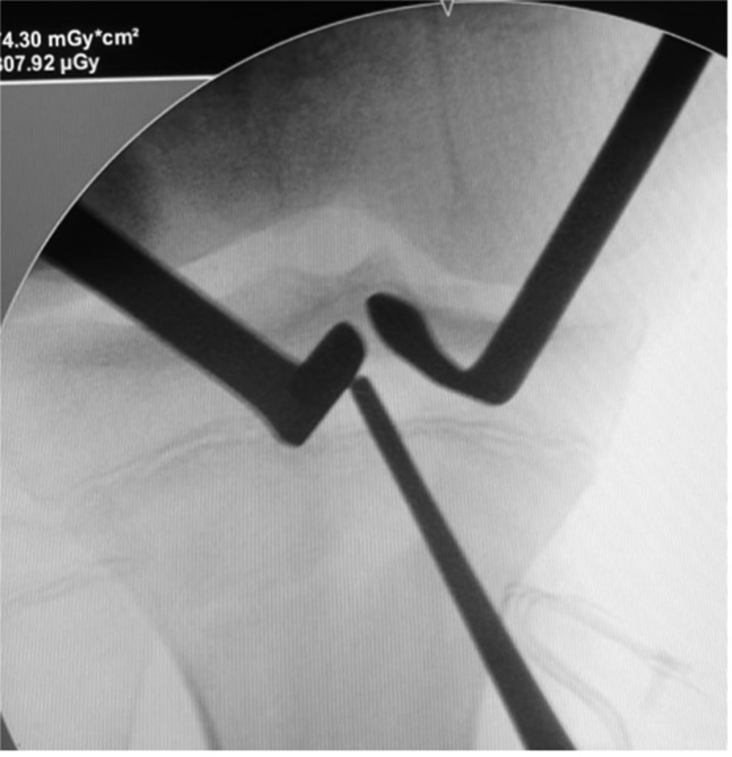
Right knee. The tibial tunnel is performed under radiologic control. A guide-pin is inserted proximal to the tibial physis.

**Fig 9:**
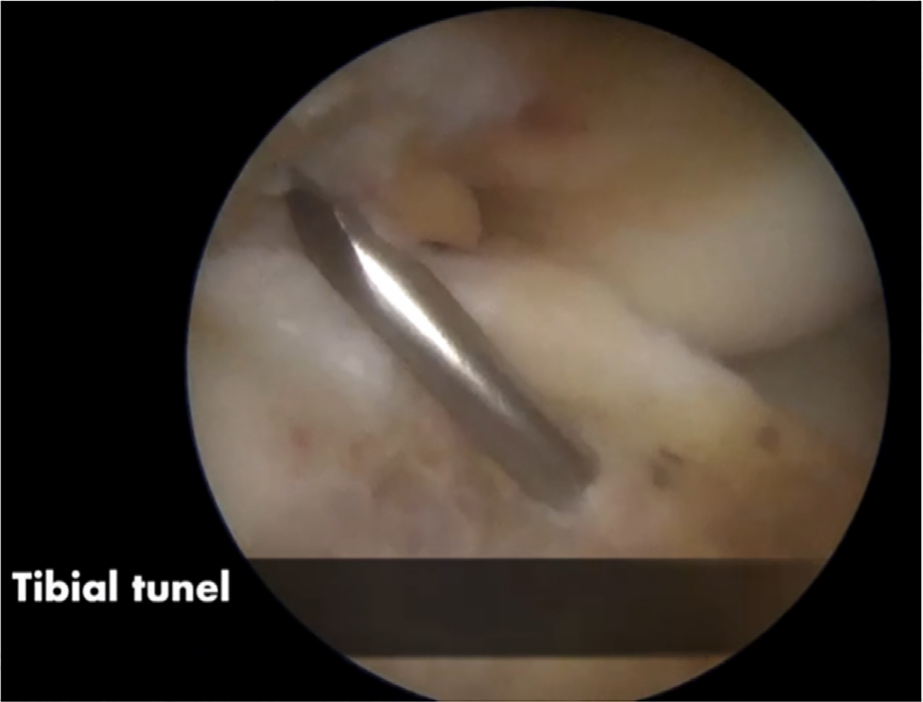
Right knee. A guide-pin is inserted over the anterior cruciate ligament tibial footprint.

**Fig 10:**
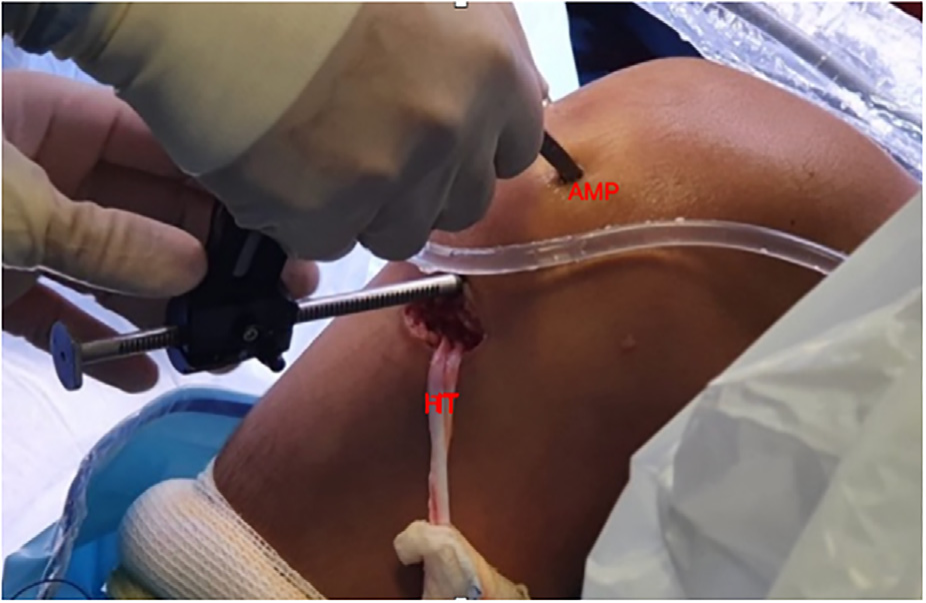
Right knee. The angulation of the tibial guide varied depending on the position of the physis. (AMP, anteromedial portal; HT, hamstring tendon.)

The tibial guide is then centered over the ACL tibial footprint under arthroscopic vision and an image intensifier (Fig 8). A guide-pin is inserted proximal to the tibial physis and drilled in an outside-in direction (Fig 9). The angulation of the tibial guide varies depending on the position of the physis (Fig 10). The diameter of the tunnel is determined by the size of the double-bundle graft (Video 1).

#### Step “Over the Top” and Graft Passage

**Fig 11:**
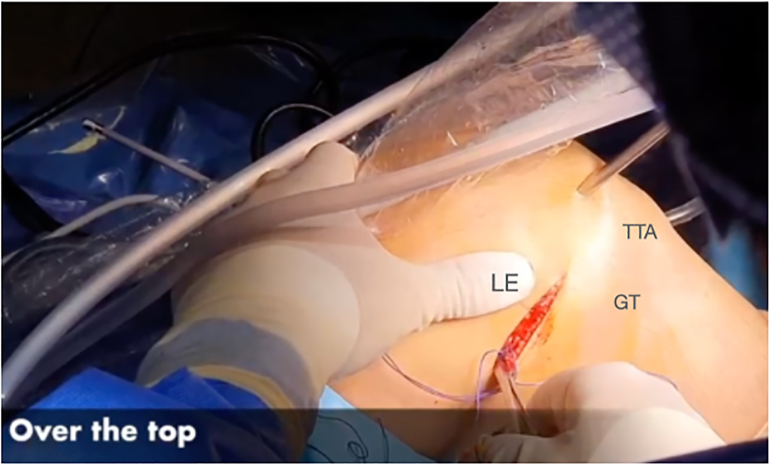
Right knee, aspect of the lateral distal femur. A small window is opened through the posterolateral capsule of the knee. (GT, Gerdy’s tubercle; LE, lateral epicondyle; TTA, tuberosity tibial anterior.)

**Fig 12:**
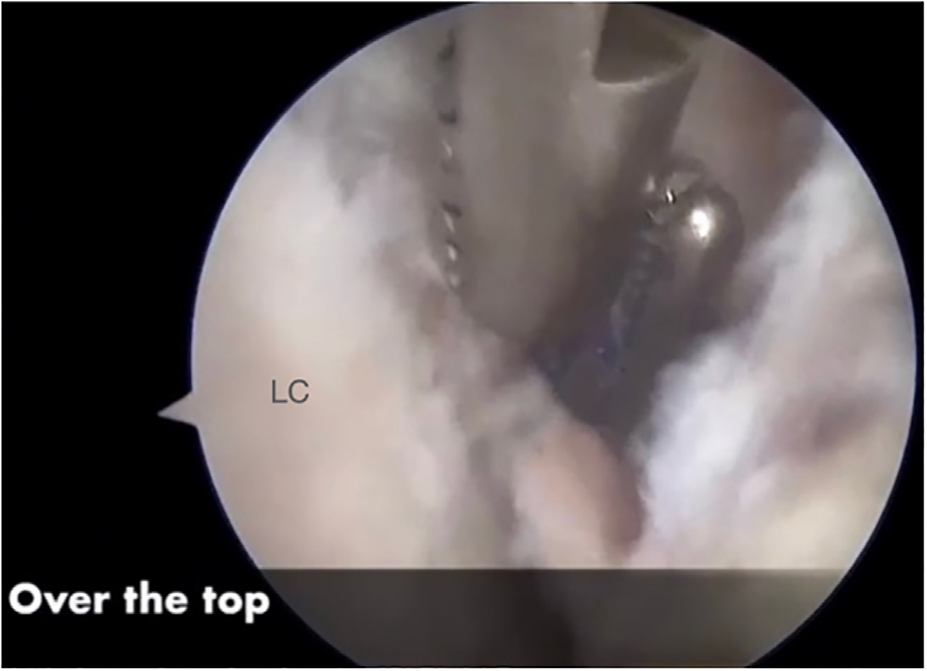
Right knee. A Kocher-type forceps is then used to pass a suture “over the top” in the lateral femoral condyle. (LC, lateral condyle.)

**Fig 13:**
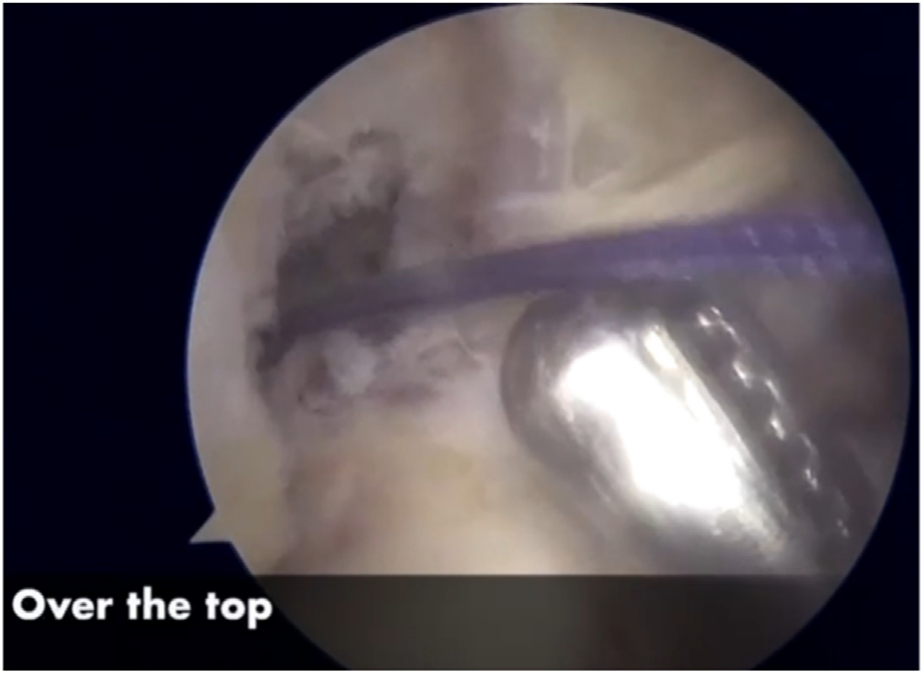
Right knee. The suture “over the top” is retrieved through the tibial tunnel. (LC, lateral condyle. TT, tibial tunnel.)

**Fig 14:**
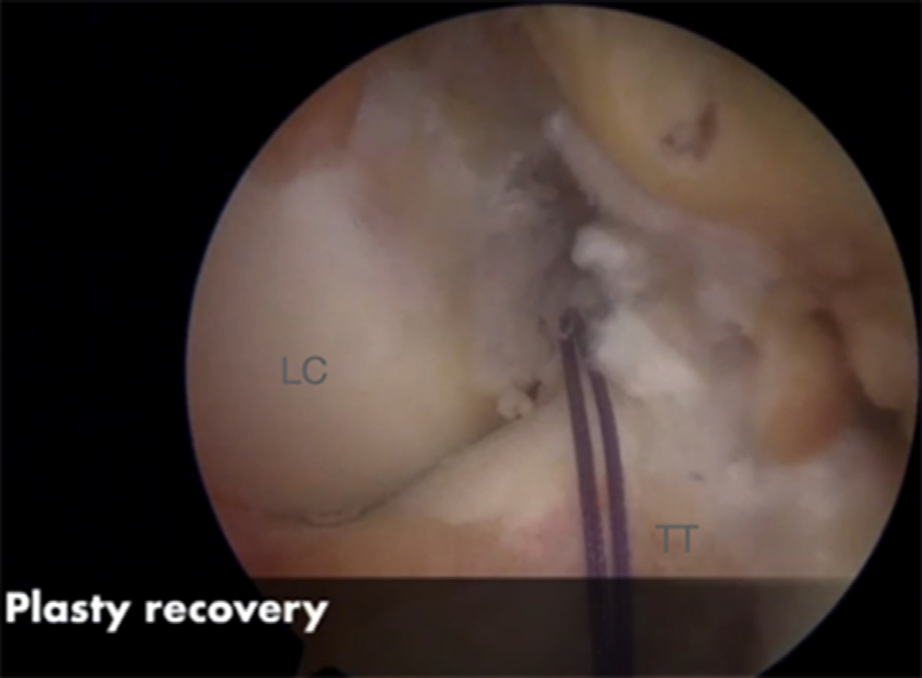
Right knee. The suture “over the top” is retrievedthrough the tibial tunnel. (LC, lateral condyle. TT, tibial tunnel.)

**Fig 15:**
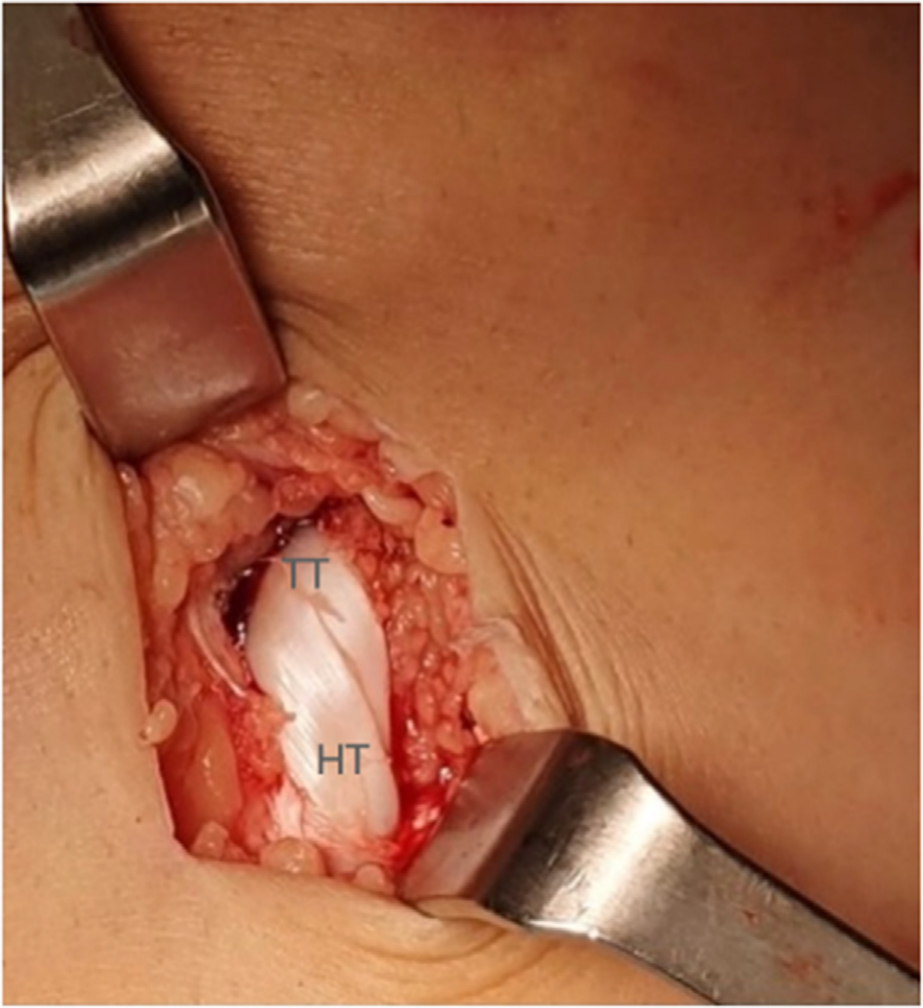
Right knee.Final appearance of the tibial tunnel graft without damaging the growth physis. TT, tibial tunnel. HT, hamstring tendons.

**Fig 16:**
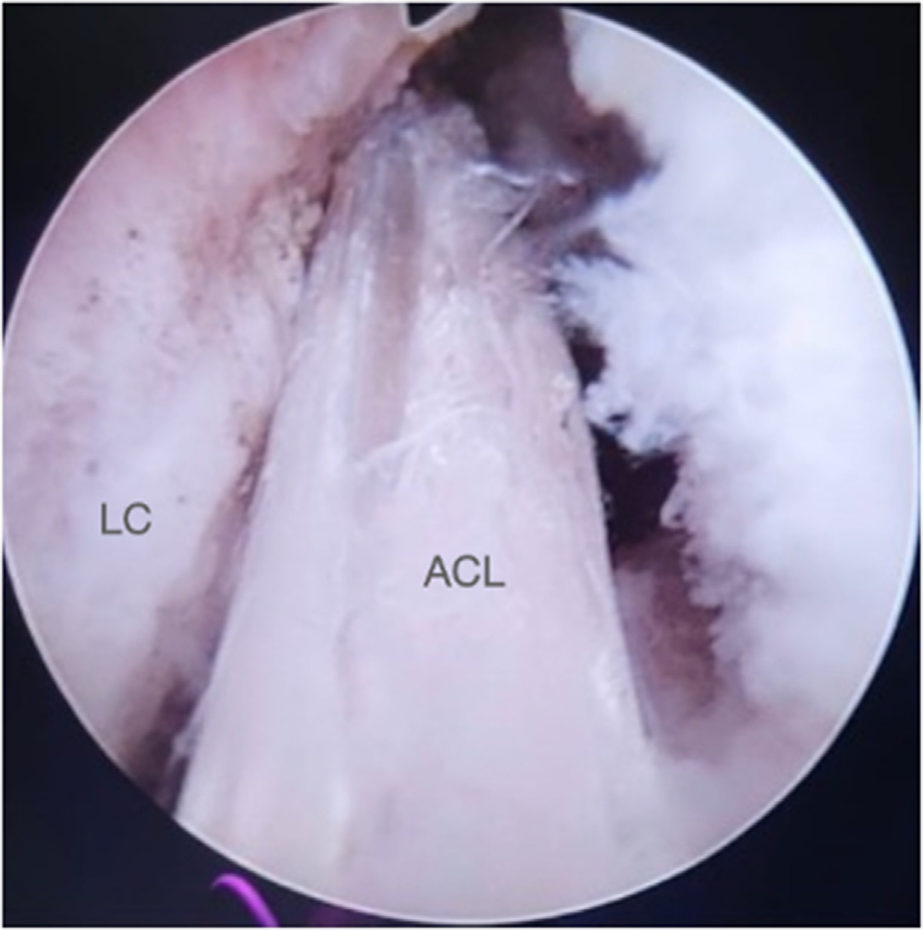
Right knee. Final appearance of the intra-articular ACL graft. (ACL, anterior cruciate ligament; LC, lateral condyle.)

**Fig 17:**
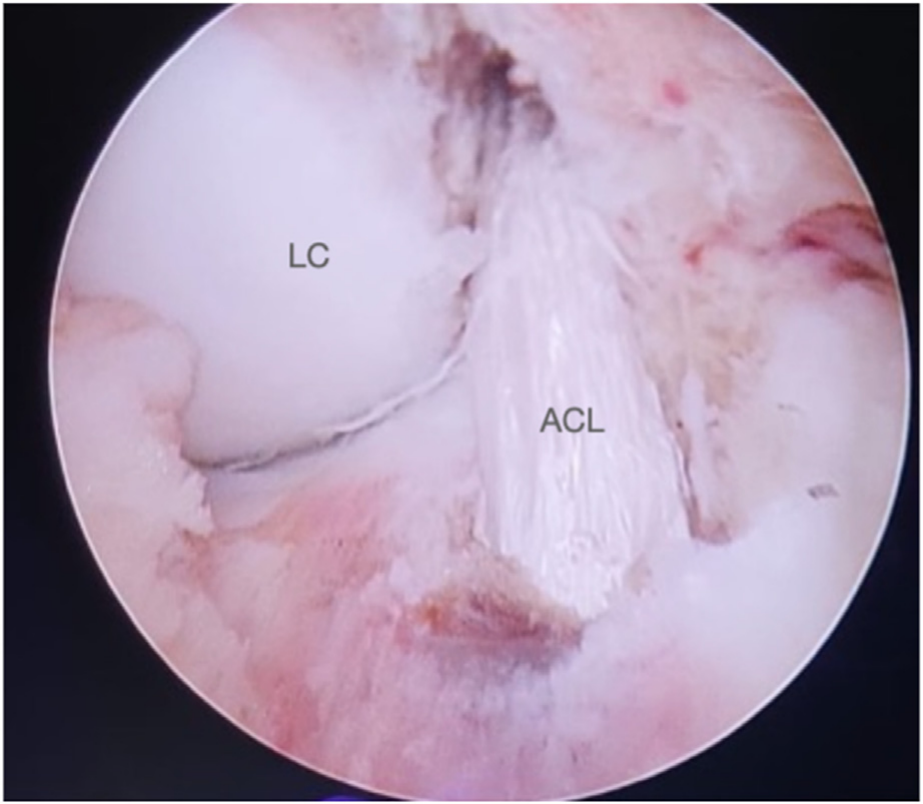
Right knee. Final appearance of the intra-articular ACL graft. (ACL, anterior cruciate ligament; LC, lateral condyle.)

Next, a small window is made through the posterolateral capsule of the knee (Fig 11). A Kocher-type forceps is then used to pass a suture “over the top” from the posterolateral window to the anteromedial portal (Fig 12). Finally, the suture is retrieved through the tibial tunnel and used to pass the graft to the posterolateral window (Fig 13, Fig 14, Fig 15, Fig 16, Fig 17, Video 1).

#### Femoral Tunnel Preparation and Graft Fixation

**Fig 18:**
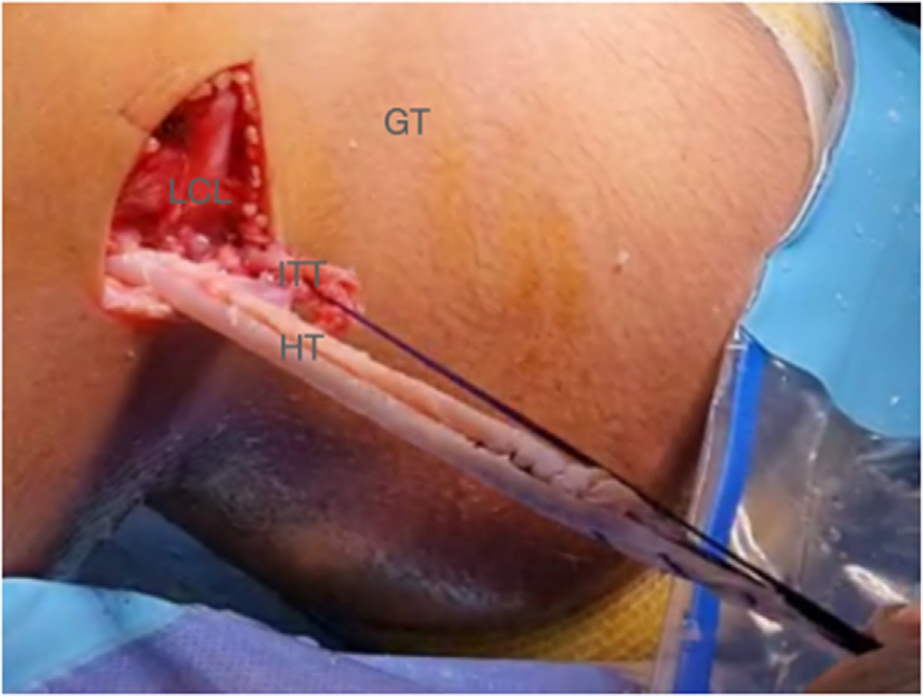
The joint caliber of the hamstring plasty and the iliotibial tract plasty is measured. (GT, Gerdy’s tubercle; HT, hamstring tendon; ITT, iliotibial tract; LCL, lateral collateral ligament.)

**Fig 19:**
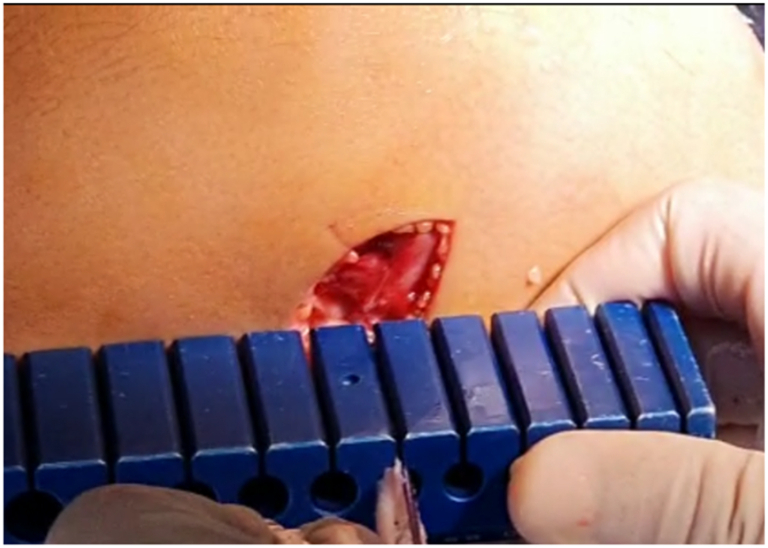
Right knee. Approach of the lateral distal femur. The joint caliber of the hamstring plasty and the iliotibial tract plasty is measured.

Next, the joint diameters of both the hamstring graft and the iliotibial tract graft are measured (Figs 18 and 19).

**Fig 20:**
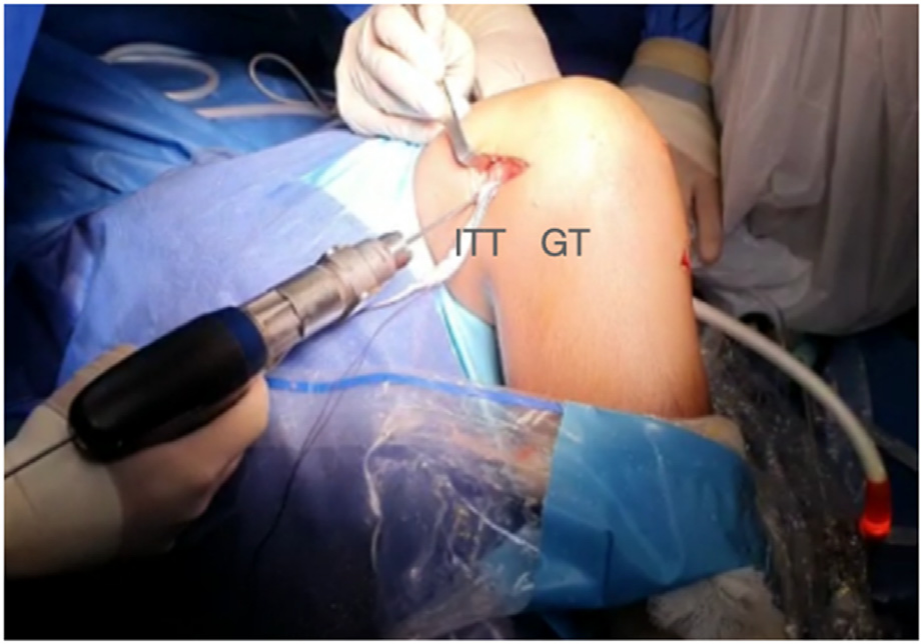
Lateral distal femur of right knee. A guide-pin is inserted proximal and posterior to the distal femoral physis from lateral to medial. (GT, Gerdy’s tubercle; ITT, iliotibial tract.)

**Fig 21:**
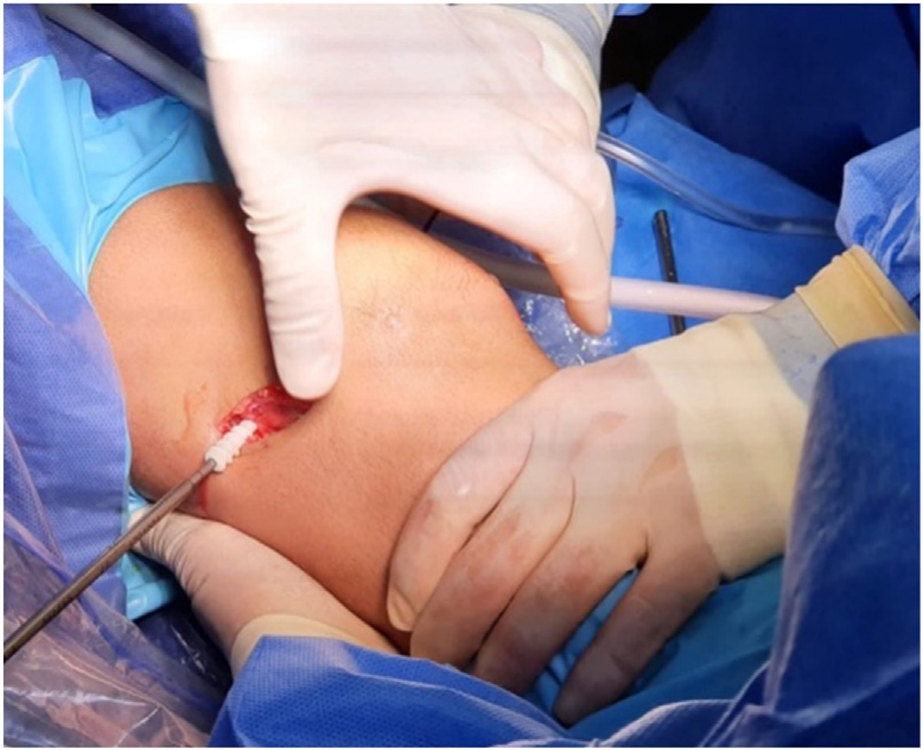
Right knee. The double-bundle graft and iliotibial tract graft are fixed in full extension and neutral rotation.

Finally, a femoral tunnel is drilled proximally and posteriorly to the distal femoral physis under image the intensifier from lateral to medial (Fig 20). The double-bundle graft and iliotibial band graft are fixed within the tunnel in full extension and neutral rotation with an interference screw (Fig 21, Video 1).

## Discussion

ACL injuries in the pediatric age group are progressively becoming more frequent due to the increase in the intensity and demand of physical activities in children.^6^ Numerous techniques and algorithms have been described for the treatment of these injuries, to avoid damage to the growth plate and to obtain higher return to play rates.^7^^,^^16^^,^^17^ For example, the LET seems to reduce ACL rerupture rate recurrence of these injuries.^18^^,^^19^

In our technique, we present a combination of an “over-the-top” ACL reconstruction and a lateral extra-articular tenodesis. All techniques for ACL reconstruction (“over the top,” transtibial, and all-epiphyseal) achieve a significant improvement in anterior-posterior and rotational stability.^8^^,^^20^ However, none of them recovers the stability levels of an intact ACL independently.^20^

Accordingly, the combination of both techniques (“over the top” and LET) could improve the biomechanical stability. Moreover, this technique combines the advantages of a physeal-sparing ACL reconstruction for patients with an immature skeleton and that of an extra-articular lateral tenodesis without the need to make 2 femoral tunnels. In addition, it eliminates the risk of femoral tunnel confluence,^21^^,^^22^ which is a real risk in pediatric patients with relatively smaller knee joints (Table 1).

**Table 1:** Advantages, Risk and Tips of the Technique

Advantages	Risks/Limitations	Tips/Pearls
Avoids drilling through the growth plate	Growth plate injury from inadequate tibial tunnel drilling	Start the surgery with the LET reinforcement to avoid subsequent inflammation.
Restores the stability by graft reconstruction	Risk of tibial plateau fracture due to angulation of the tibial tunnel	Start the skin incision for graft harvesting just above the line of insertion to facilitate perforation of the tibial epiphyseal tunnel.
Minimizes the risk of cartilage damage from recurrent knee instability	Surgery through the periosteum near the tibial physis may also stimulate growth.	Perform full femoral tunnel to avoid problems of tension of the plasty.
Minimizes the risk of confluence of femoral tunnels	Plasty thickness less than 8 mm	Fix the graft in 0° of extension and neutral rotation.
Maintains the vascularization and innervation of the gracilis and semitendinosus tendons		Use the tibial angulation necessary to avoid damaging the tibial growth plate.
The decreased diameter of the tibial tunnel increases the safety margin for keeping the tunnel within the tibial epiphysis and avoids the risk of fracture.		
No plasty length issues		

LET, extra-articular lateral tenodesis.

Similar techniques have been described in the literature. Marcacci et al.^23^ combined an ACL reconstruction using an “over-the-top” technique with an extra-articular lateral reinforcement. In contrast to our technique, Marcacci et al.^23^ used the remaining hamstring graft and fixed it with a staple at the anterolateral ligament’s origin in the tibia. Accordingly, this technique does not need any femoral tunnels, but it is dependent on the length on the tendinous remnant.

In our technique, the iliotibial band was used to perform the lateral tenodesis. Therefore, we did not rely on the total hamstring graft length. Moreover, by using an interference screw fixation, we avoided any potential discomfort caused by the staples.

Grassi et al.^24^ and Zaffagnini et al.^8^ reported good clinical results and low rerupture rates using this technique with 10 and 20 years of follow-up, respectively. Moreover, the authors did not report an increase in lateral compartment osteoarthritis.

The main difference between our technique and the modified Lemaire-type extra-articular lateral tenodesis lies in the femoral fixation point. The femoral location of this tenodesis has been analyzed in various anatomic and biomechanical studies. Accordingly, it has been described to be proximal and posterior to the epicondyle.^25^

In our case, the position of the physis should be taken into consideration when fixing the tenodesis, placing it as proximal as necessary so as not to invade it.

As Kittl et al.^26^ demonstrated, the most important thing in performing this technique is passing the tenodesis under the lateral collateral ligament, acting as a pulley. The fixation must be proximal to the lateral epicondyle, regardless of the exact point once the plasty has passed under the lateral collateral ligament.

Both the Lemaire technique and ACL reconstruction can be fixed in full extension, therefore making this technique suitable for simultaneous fixation. However, a drawback of our technique could be the thickness of the ACL graft, which usually does not measure more than 6 mm in diameter. Some authors have described that the tunnel localization is a more relevant factor in ACL reconstruction failure than the diameter of the ACL graft.^27^ Furthermore, in previous studies, Di Sarsina et al.^7^ and Grassi et al.^28^ reported excellent results in skeletally immature patients subjected to an ACL reconstruction using the technique by Marcacci et al.^23^ with graft diameters similar to the ones found in our study.

The preservation of the tibial insertion of the gracilis and semitendinous tendons maintains their vascularization and innervation, therefore improving their integration compared with free grafts.^29^^,^^30^

## Conclusions

The “over-the-top” technique for ACL reconstruction with an extra-articular lateral tenodesis is a safe technique that may increase the rotational stability of the operated knee, avoiding the confluence of tunnels and the risk of rupture of the plasty.

## Supplementary Data

ICMJE author disclosure forms

Video 1 The patient is placed in a supine position with a tourniquet on the proximal thigh. An L-shaped support is placed on the distal end of the table to keep the knee at about 100° of flexion. Two additional supports are placed laterally. An exploration of the knee joint is performed under general anesthesia showing a positive Lachman test. A 3- to 4-cm lateral incision is performed over the lateral epicondyle, and a 1-cm-thick band is detached from the iliotibial band and extended from Gerdy’s tubercle proximally. The free end of the graft is sutured with a high-strength suture and passed under the lateral collateral ligament (LCL). Then, an oblique incision is made on the medial aspect of the tibia. The gracilis and semitendinous tendons are then harvested using a tenotome, without releasing their distal insertions. Next, the tendons are cleaned from any remaining muscle fibers, and their ends are sutured with a high-strength suture. The diameter of the double-bundle autograft is measured to create the tibial tunnel. Standard anterolateral and anteromedial portals are made to perform a full exploration of the knee. The anterior cruciate ligament (ACL) tear is confirmed and a debridement of any remnant tissues is performed using an arthroscopy shaver. The exit point of the tibial tunnel is marked with an electrocautery device*.* The tibial guide is then centered over the ACL tibial footprint under arthroscopic vision and an image intensifier. A guide-pin is inserted proximally to the tibial physis and drilled in an outside-in direction. The angulation of the tibial guide varies depending on the position of the physis. The diameter of the tunnel is determined by the size of the double-bundle graft. Next, a small window is made through the posterolateral capsule of the knee. A Kocher-type forceps is then used to pass a suture “over the top” from the posterolateral window to the anteromedial portal. The suture is then retrieved through the tibial tunnel and used to pass the graft to the posterolateral window. Next, the diameters of the hamstring graft and the iliotibial band graft are measured*.* Finally, a femoral tunnel is drilled proximally and posteriorly to the distal femoral physis under the image intensifier from lateral to medial. The double-bundle graft and iliotibial tract graft are fixed within the tunnel in full extension and neutral rotation with an interference screw.
